# Germline sequence variants in *TGM3* and *RGS22* confer risk of basal cell carcinoma

**DOI:** 10.1093/hmg/ddt671

**Published:** 2014-01-08

**Authors:** Simon N. Stacey, Patrick Sulem, Daniel F. Gudbjartsson, Aslaug Jonasdottir, Gudmar Thorleifsson, Sigurjon A. Gudjonsson, Gisli Masson, Julius Gudmundsson, Bardur Sigurgeirsson, Kristrun R. Benediktsdottir, Kristin Thorisdottir, Rafn Ragnarsson, Victoria Fuentelsaz, Cristina Corredera, Matilde Grasa, Dolores Planelles, Onofre Sanmartin, Peter Rudnai, Eugene Gurzau, Kvetoslava Koppova, Kari Hemminki, Bjørn A Nexø, Anne Tjønneland, Kim Overvad, Hrefna Johannsdottir, Hafdis T. Helgadottir, Unnur Thorsteinsdottir, Augustine Kong, Ulla Vogel, Rajiv Kumar, Eduardo Nagore, José I. Mayordomo, Thorunn Rafnar, Jon H. Olafsson, Kari Stefansson

**Affiliations:** 1 deCODE Genetics/AMGEN, Sturlugata 8, 101 Reykjavik, Iceland; 2 Landspitali University Hospital, IS-101 Reykjavik, Iceland; 3 Faculty of Medicine, University of Iceland, IS-101 Reykjavik, Iceland; 4 Division of Dermatology, Gregorio Marañón Hospital, Madrid 28007, Spain; 5 Division of Dermatology, University Hospital, Zaragoza 50009, Spain; 6 Laboratory of Histocompatibility-Molecular Biology, Centro de Transfusión de la Comunidad Valenciana, Avenida del Cid, 65-A, Valencia 46014, Spain; 7 Department of Oncology, Instituto Valenciano de Oncologia, Valencia 46009, Spain; 8 Universidad Católica de Valencia, Valencia, Spain; 9 Department of Environmental Epidemiology, National Institute of Environmental Health, Budapest H-1450, Hungary; 10 Health Department, Environmental Health Centre, Babes Bolyai University, Cluj-Napoca, Romania; 11 Department of Environmental Health, Regional Authority of Public Health, Banska Bystrica SK-975 56, Slovakia; 12 Division of Molecular Genetic Epidemiology, German Cancer Research Centre, Heidelberg D-69120, Germany; 13 Department of Biomedicine and; 14 Department of Public Health, Institute of Epidemiology and Social Medicine, University of Aarhus, DK-8000 Aarhus C, Denmark; 15 Danish Cancer Society Research Centre, DK-2100 Copenhagen Ø, Denmark; 16 National Research Centre for the Working Environment, DK-2100 Copenhagen, Denmark; 17 University of Zaragoza, Zaragoza 50009, Spain

## Abstract

To search for new sequence variants that confer risk of cutaneous basal cell carcinoma (BCC), we conducted a genome-wide association study of 38.5 million single nucleotide polymorphisms (SNPs) and small indels identified through whole-genome sequencing of 2230 Icelanders. We imputed genotypes for 4208 BCC patients and 109 408 controls using Illumina SNP chip typing data, carried out association tests and replicated the findings in independent population samples. We found new BCC susceptibility loci at *TGM3* (rs214782[G], *P* = 5.5 × 10^−17^, OR = 1.29) and *RGS22* (rs7006527[C], *P* = 8.7 × 10^−13^, OR = 0.77). *TGM3* encodes transglutaminase type 3, which plays a key role in production of the cornified envelope during epidermal differentiation.

## INTRODUCTION

Cutaneous basal cell carcinoma (BCC) is the most common cancer of humans. While it is rarely metastatic, it can be locally invasive and can cause considerable morbidity and economic burden (1). In common with other forms of skin cancer, the most significant environmental risk factor is UV exposure, but both high- and low-penetrance sequence variants also affect risk (2–8). Sometimes the affected genes can be linked to endogenous factors determining reactions to UV exposure (9). The way in which some other variants act to promote BCC susceptibility is more obscure.

Previously we used whole-genome sequencing and imputation to search for variants associated with predisposition to BCC (8). In this study, we have increased the sample sizes and the number of DNA sequence variants examined, to search for new variants predisposing to BCC. Variants were identified by whole-genome sequencing of 2230 Icelanders to an average coverage of at least 10×. We detected ∼38.5 million single nucleotide polymorphisms (SNPs) and small indels. We used imputation assisted by long-range haplotype phasing and genealogy-based *in silico* genotyping to determine the genotypes of these variants for 4208 Icelanders with BCC and 109 408 controls (8,10–12). We report on the discovery of two new BCC predisposition loci: *TGM3* and *RGS22*. Variants at these loci passed the Bonferroni-adjusted *P*-value threshold of genome-wide significance in Iceland: ∼1.2 × 10^−9^. The associated variants have minor allele frequencies (MAFs) of 0.17 and 0.14, respectively. We did not observe any new rare or low frequency variants (<0.02 MAF) that passed the Bonferroni threshold of significance in the Icelandic samples. Nevertheless, the availability of association data derived from whole-genome sequence information allows for fine mapping of loci in tandem with their discovery.

## RESULTS

We first examined BCC susceptibility loci that we had previously identified in GWAS studies, in order to validate our approach and to gain further evidence for the published associations. As shown in Supplementary Material, Table S1, all of the previously published association results were confirmed to greater levels of significance. In each case, the odds ratio point estimate was slightly diminished, probably as a result of a “winners’ curse” tending to bias the original point estimates upwards (13,14). We also noted that two variants, in *TGM3* and *RGS22*, which had been observed at suggestive levels of significance in our previous analysis (8), now achieved the Bonferroni-adjusted level of genome-wide significance.

**Figure 1.**
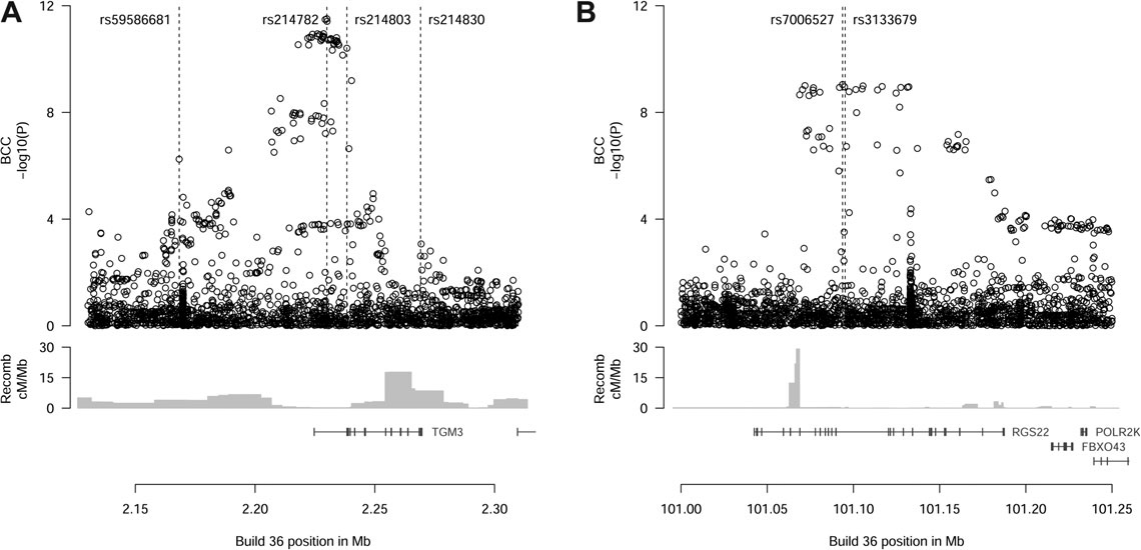
Association signals at the (**A**) TGM3 and (**B**) RGS22 loci. The upper panels show the BCC association signals [expressed as −log_10_(*P*)] for variants identified by whole-genome sequencing and imputation. Positions of key single nucleotide polymorphisms discussed in the text are indicated. The middle panel shows recombination rates calculated as described previously (39). The lower panel shows the locations of RefSeq genes in the region.

As shown in Figure 1A, the current analysis revealed a cluster of variants in and near the 5′ end of the *TGM3* gene that was associated with risk of BCC. The strongest signal originated from rs214782[G] (*P* = 3.1 × 10^−12^, OR = 1.29)(Table 1). Also in the cluster was a missense variant rs214803 T13K. The linkage disequilibrium (LD) between rs214782 and rs214803 is *r*^2^ = 0.967, D′ = 0.995 in the Icelandic data (see also Supplementary Material, Table S2). In order to confirm the association results, we generated Centaurus (15) single-track genotyping assays for rs214782 (*Top*) and rs214803 (*T13K*). First, we used them to confirm the imputation accuracy in the Icelandic population (Supplementary Material, Table S3). We then used them to investigate the associations in case–control sample sets from Spain, Eastern Europe and Denmark. The evidence for replication of the BCC association was significant and showed no evidence of heterogeneity in the non-Icelandic populations. Combined with the Icelandic data, the overall association is highly significant (*P* = 5.5 × 10^−17^, OR = 1.29 for rs214782; Table 1, Supplementary Material, Table S4). Adjustment for age (at diagnosis for cases, at sampling for controls) had no effect on the association (Supplementary Material, Table S5). Accordingly, we concluded that *TGM3* is a BCC susceptibility locus.

**Table 1.** Association of SNPs in TGM3 and RGS22 with BCC

SNP	Allele	Chr	Position^a^	Locus	Description in text	Sample set	Number cases	Number controls	Frequency in controls	OR	95% CI	*P*
rs214782	G	20	2 229 970	TGM3	Top	Iceland	4208^b^	109 408^c^	0.17	1.29	(1.20, 1.38)	3.1 × 10^−12^
						Combined non-Icelandic	1480	4409		1.31	(1.17, 1.46)	3.5 × 10^−6^
						All combined	5688	113 817		1.29	(1.22, 1.37)	5.5 × 10^−17^
rs214803	G	20	2 238 333	TGM3	T13K	Iceland	4208^b^	109 408^c^	0.17	1.27	(1.18, 1.37)	3.9 × 10^−11^
						Combined non-Icelandic	1434	4610		1.32	(1.18, 1.48)	2.2 × 10^−6^
						All combined	5642	114 018		1.28	(1.21, 1.37)	5.0 × 10^−16^
rs59586681	T	20	2 168 310	TGM3	Distal	Iceland	4208^b^	109 408^c^	0.39	0.86	(0.81, 0.91)	5.7 × 10^−7^
						Combined non-Icelandic	1454	4386		0.85	(0.77, 0.94)	0.0012
						All combined	5662	113 794		0.86	(0.82, 0.90)	2.5 × 10^−9^
rs214830	G	20	2 269 105	TGM3	G654R	Iceland	4208^b^	109 408^c^	0.31	0.91	(0.85, 0.97)	0.0024
						Combined non-Icelandic	1466	4543		0.94	(0.83, 1.05)	0.27
						All combined	5674	113 951		0.91	(0.87, 0.97)	0.0014
rs7006527	C	8	101 093 681	RGS22	Top	Iceland	4208^b^	109 408^c^	0.14	0.77	(0.70, 0.83)	9.0 × 10^−10^
						Combined non-Icelandic	1427	4442		0.77	(0.67, 0.88)	2.3 × 10^−4^
						All combined	5635	113 850		0.77	(0.71, 0.82)	8.7 × 10^−13^

^a^NCBI HG18 Build 36.

^b^Total number of cases used for association testing, including 2726 chip-genotyped and 1482 *in silico-*genotyped individuals.

^c^Total number of controls used for association testing, including 70 876 chip-genotyped and 38 532 *in silico*-genotyped individuals.

The rs214803 (*T13K*) missense variant is predicted by SIFT to be “tolerated” (score 0.29) and by PolyPhen to be “benign” (score 0.018). Moreover, the signal from rs214803 (*T13K*) was not significant when adjusted for the effect of rs214782 (*Top*), whereas rs214782 (*Top*) remained significant when adjusted for rs214803 (*T13K*)(Supplementary Material, Table S6). It is therefore quite conceivable that the pathogenic effect underlying the main signal is exerted by rs214782 (*Top*) or one of the other non-coding variants in high LD with it. Having whole-genome sequence data meant that we could examine a fairly complete set of such variants. Using conditional analysis, we determined a set of correlated SNPs and small indels whose effects were statistically indistinguishable from rs214782 (*Top*) at a significance cut-off of *P* = 10^−4^ (see Materials and methods). A set of 40 such variants were identified, which included rs214803 (*T13K*) (Supplementary Material, Table S7).

We then examined these variants for sequence overlap with potential regulatory sites by cross-referencing them to ENCODE data (16,17). Five of the 40 variants are in regulatory regions identified by Ensembl with an ENSR number (Supplementary Material, Table S7). Both the Top SNP rs214782 and a highly correlated (*r*^2^ = 0.997) variant rs214783 are located in a DNAseI hypersensitive site with ChIP-Seq evidence of binding several transcription factors in different cell lines, including members of the Jun and Fos families. Another highly correlated SNP (rs214799, *r*^2^ = 0.967) occurs at a site with evidence of binding FOXA1 and FOXA2 (Supplementary Material, Table S7).

**Figure 2.**
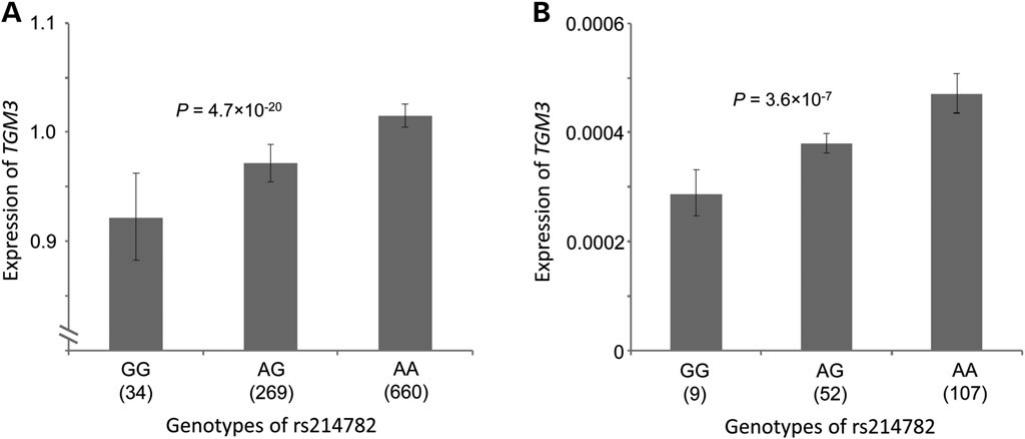
The BCC risk allele rs214782[G] is associated with reduced expression of TGM3 in blood-derived RNA. (**A**) Expression of TGM3 RNA for three genotypes of rs214782, measured in RNA from whole blood samples from 963 individuals using Agilent microarrays. The expression is shown as 10^(average MLR)^ where MLR is the mean log expression ratio and the average is over individuals with the indicated genotype. The vertical bars indicate the s.e.m. Significance was determined by regressing the MLR values against the number of risk alleles that each individual carries, adjusting for age, sex, familial relatedness and differential cell count in blood. (**B**) For confirmation, a subset of 168 RNA samples from (A) were tested using RT-PCR and analysed similarly.

We examined rs214782 for an effect on nearby gene expression (*cis*-eQTL) using microarray data that we derived previously from blood and adipose tissue (18). Even though *TGM3* is primarily expressed in epidermis, we were able to detect a strong *cis*-eQTL with rs214782 in blood (*P* = 4.7 × 10^−20^, Fig. 2A). No other variant that we detected within a 1-Mb window had a substantially more significant eQTL. We confirmed the effect of rs214782 on *TGM3* expression by RT-PCR (Fig. 2B). Note that increased risk of BCC is associated with the low-expressor [G] allele of rs214782. Thus, it appears that an effect on *TGM3* gene expression is as likely as the T13K coding variant to account for the BCC susceptibility at this locus.

We noted that a variant located 5′ to the *TGM3* gene and with an MAF approaching 0.40 showed a protective effect (OR = 0.86, *P* = 5.7 × 10^−7^ in Iceland, Table 1). This variant, designated rs59586681 (*Distal*), is separated from rs214782 (*Top*) by a region of moderate recombination (Fig. 1), and the two variants are not well correlated (*r*^2^ = 0.01, D′ = 0.21 in Iceland, see also Supplementary Material, Table S2). Conditional analysis of the Icelandic data showed that rs59586681 (*Distal*) retains a nominally significant signal once the effect of rs214782 (*Top*) is taken into account (*P*_adj_ = 1.8 × 10^−4^, OR_adj_ = 0.90). The rs59586681 (*Distal*) association replicated significantly in the non-Icelandic population samples, both with and without adjustment for the effect of rs214782 (*Top*)(Table 1, Supplementary Material, Table S6). This suggests that more than one pathogenic variant is present at the *TGM3* locus.

Because rs214803 (*T13K*) is potentially pathogenic, we searched *TGM3* for other coding variants that might be associated with BCC. In addition to T13K, sequence analysis uncovered seven missense variants within *TGM3* for which imputation and association analysis were possible. One of these, rs214830 (*G654R*) in the last exon of the gene, is quite common in European populations (0.31 MAF in Iceland). It showed a nominally significant protective effect on BCC: *P* = 0.0024, OR = 0.91 (Table 1). As might be expected from the large recombination rate peak separating rs214830 (*G654R*) from the other signal SNPs (Fig. 1), the association with BCC persisted after adjustment for the effects of rs214782 (*Top*) and rs59586681 (*Distal*) both individually and jointly (Supplementary Material, Table S6). In the non-Icelandic samples, the effect of rs214830 (*G654R*) was consistent with Iceland but not independently significant. Overall rs214830 (*G654R*) reached a significance level of *P* = 0.0014 (Table 1).

The G654 variant is predicted by SIFT to be “tolerated” (score = 1) and “benign” by PolyPhen (score = 0). A correlated variant occurs in the 3′ UTR of *TGM3* (rs214831, *r*^2^ = 0.81, D′= 0.92), and it gave a similar association signal in Iceland: *P* = 8.52 × 10^−4^, OR = 0.901. In a conditional analysis, the effects of these two variants could not be distinguished. Therefore, the two variants are equally likely to be responsible for the observed pathogenic effect.

At the second genome-wide significant locus, we observed a cluster of intronic signals in *RGS22* (Fig. 1B). The strongest signal came from rs7006527 (OR = 0.77; *P* = 9 × 10^−10^) with an MAF of ∼0.14 in controls. Using a single-track Centaurus assay for rs7006527, we confirmed the imputed results in Iceland (Supplementary Material, Table S3) and genotyped the foreign population samples. As shown in Table 1, the signal replicated significantly outside Iceland (*P* = 2.3 × 10^−4^, OR = 0.77, see also Supplementary Material, Table S4). The overall association including Iceland and replication cohorts was highly significant (*P* = 8.7 × 10^−13^, OR = 0.77). Adjustment for age had no effect on the association (Supplementary Material, Table S5).

We carried out a conditional analysis to determine whether there are other detectable signals present at the *RGS22* locus. After adjustment for the effect of rs7006527, we observed a signal from a group of 42% MAF variants typified by rs3133679 (*P*_adj_ = 7.0 × 10^−6^, OR_adj_ = 0.88, Supplementary Material, Table S6). Interestingly, the signal from this group of variants was practically undetectable in the absence of adjustment for rs7006527 (*P* = 0.020, OR = 0.94 for rs3133679 unadjusted). After adjusting for rs7006527 and rs3133679 jointly, no substantial signals remained at the *RGS22* locus. We typed rs3133679 in the foreign replication samples and adjusted the results for the effect of rs7006527. Although rs3133679 replicated nominally in the foreign samples without adjustment for rs7006527, no significant *P*_adj_ signal was observed for rs3133679. Thus, although the *P*_adj_ for rs3133679 was significant over all populations studied, the effect was entirely attributable to the signal from the Icelandic population (Supplementary Material, Table S6).

Using the method described earlier for *TGM3*, we found a set of 24 variants at the *RGS22* locus, which are statistically indistinguishable from rs7006527 (Supplementary Material, Table S8). All were in *RGS22* introns. Only one had been assigned an ENSR identifier by Encode, and it lacked ChIP-Seq evidence of transcription factor binding. There was no evidence of an eQTL associated with rs7006527, nor was any significant eQTL affecting *RGS22* expression observed within a 1-Mb window.

## DISCUSSION

In summary, we have found two new loci associated with BCC susceptibility. The associations at both loci appear to be specific to BCC, as we have not yet observed any significant associations with other cancers. Moreover, they do not seem to affect the pigmentation traits sometimes associated with BCC susceptibility variants (3).

*RGS22* is a little-characterized, putative regulator of G-protein signalling that is normally expressed only in testis (19). It is reported to interact with guanine nucleotide binding proteins GNA12, GNA13 and GNA11. The protein may be ectopically expressed in some types of invasive carcinoma (20). The mechanism behind the effect of *RGS22* variants on BCC susceptibility remains to be elucidated.

*TGM3* encodes type 3 transglutaminase (TGase-3), an enzyme with Ca^2+^-dependent transamidation activity in the non-proliferating layers of the epidermis and in hair follicles (21). Along with TGase-1 and TGase-5, the principle function of TGase-3 in epidermis is the generation of protein–protein crosslinks through the formation of isopeptide bonds between peptidyl glutamine and lysine residues (22). In the outer layers of the epidermis, keratinocytes terminally differentiate to form the cornified layer or stratum corneum. This consists of flattened, dead cells called corneocytes embedded in a lamellar lipid–protein matrix. During formation of the corneocytes, a set of structural proteins including involucrin, loricrin, periplakin and desmoplakin and small proline-rich proteins (SPRs) are cross-linked below the plasma membrane to form a resilient structure known as the cornified envelope (CE). The CE gives the cornified layer flexible mechanical resistance and provides a scaffold for the formation of intercellular corneodesmosome links and of the extracellular lamellar lipid–protein matrix (23). In the initial formation of the CE, involucrin, periplakin and desmoplakin are cross-linked by TGase-1 and TGase-5 to form a monomolecular scaffold layer beneath the plasma membrane. Subsequently, loricrin and SPRs are oligomerized by TGase-3, which are then cross-linked into the scaffold. The CE is subsequently linked to the keratin intermediate filament network and, through the actions of TGase-1, to components of the lamellar lipid matrix. In hair follicles, TGase-3 probably functions to cross-link structural proteins of the hair fibre (24).

TGase-1 is indispensable for normal formation of epidermis. In mice, *Tgm1* knockouts die perinatally owing to dehydration from a defective cornified layer and consequent skin barrier dysfunction (25). Human mutations in *TGM1* cause type 1 autosomal recessive congenital icthyosis, characterized by a scaly skin surface, epidermal hyperplasia and barrier defects (23). On the other hand, loss of TGase-3 has much more subtle effects. *Tgm3* knockout mice exhibit an *in utero* developmental delay in skin barrier formation, but the cornified layer is normalized by the time of birth and the mice do not suffer from perinatal dehydration. They do display abnormalities of hair follicle function (26). In humans, genetic variants in *TGM3* have not been associated previously with disease. Autoantibodies against TGase-3 are prevalent in the cutaneous blistering condition dermatitis herpetiformis (27). Loss of *TGM3* expression has been reported to have prognostic significance in oesophageal cancer (28).

How might a variant in *TGM3* contribute to susceptibility to BCC? One possibility is that compromised TGase-3 activity might disrupt the normal differentiation and cell death programme of corneocytes. The cell death mechanism in corneocytes is neither apoptotic nor necrotic, but is a special type of programmed cell death involving complete disintegration of subcellular organelles and maturation of the CE. Orderly cell death by cornification prevents intracellular contents from being spilled in uncontrolled or necrotic cell death, which could release DAMPs (danger associated molecular patterns) and set off an inflammatory response within the epidermis (29). Inflammation in epidermis will lead to epidermal hyperplasia and creates a tumour-promoting environment (30). Secondly, defective formation of the CE might compromise skin barrier function. Epidermal hyperplasia is an invariable homeostatic response to a failure of barrier function (23). Studies in mice have implicated epidermal integrity, inflammatory responses and hyperplasia as key pathways in skin carcinogenesis (31–33). Thus, we speculate that a genetic variant in TGM3 causing a mild but chronic disturbance of corneocyte differentiation and an ongoing barrier defect could increase susceptibility to BCC.

## MATERIALS AND METHODS

### Subjects

#### Iceland

Approval for the study was granted by the Icelandic National Bioethics Committee and the Icelandic Data Protection Authority. Affected individuals were identified through the Icelandic Cancer Registry (ICR), which has maintained records of BCC diagnoses since 1981. The records contained only cases of histologically verified BCC, sourced from all the pathology laboratories in the country that deal with these lesions. Icelandic controls consisted of individuals selected from other ongoing association studies at deCODE and who did not have a diagnosis of BCC recorded in the ICR. Median age at diagnosis for cases was 66 years (range 10–104). All subjects were of European ancestry.

#### Eastern Europe

Details of this case–control set have been published previously (34). Briefly, BCC cases were recruited from all general hospitals in three study areas in Hungary, two in Romania and one in Slovakia. Cases were identified on the basis of histopathological examinations by pathologists. The median age at diagnosis was 67 years (range 30–85). Controls were recruited from the same hospitals. Individuals with malignant disease and diabetes were excluded. Local ethical boards approved of the study. All subjects were of self-reported European ancestry.

#### Spain Valencia

Eligible participants were recruited from the outpatient dermatology clinics of the Instituto Valenciano de Oncología in Valencia, Spain, starting from May 2003. Cases were patients with histologically proven BCC presenting with superficial or nodular lesions of <1 cm in diameter. Clinical and pathological data from these patients are prospectively collected by medical history review, personal interview and clinical examination by an expert dermatologist. Immunocompromised patients were excluded as were those with any autoimmune disease, hereditary disorders that include the presence of BCC (Gorlin syndrome and xeroderma pigmentosum) and epidermodysplasia verruciformis. Controls were disease-free and ethnically matched healthy subjects recruited at the Transfusion Centre of Valencia. Phenotypic characteristics of controls were obtained by a self-administered structured questionnaire. All subjects were of self-reported European ancestry. All patients in the study had signed an informed consent, and the study protocol was approved by the institutional ethics boards.

#### Spain Zaragoza

BCC cases were recruited from the Oncology Department of Zaragoza Hospital starting from September 2007. Individuals with histologically proven invasive BCC were eligible to participate in the study. All subjects were of self-reported European ancestry. The median time interval from BCC diagnosis to collection of blood samples was 14 months (range 1–53 months). Median age at diagnosis was 69 years (range 21–91).

#### Denmark

Subjects were participating in the “Diet, Cancer and Health” study, which is a prospective study of 57 053 individuals recruited at two centres in Denmark between December 1993 and May 1997 (35). Subjects were monitored during the follow-up period, and BCC cases were identified through linkage to entries in the Danish Cancer Registry. Each case identified was matched to one control from the Diet, Cancer and Health cohort based on gender, age on study entry and age at diagnosis of the case. Median age at diagnosis was 59 (range 50–68). In addition to extensive diet and lifestyle questions, subjects answered questions regarding their skin sensitivity to sun, tanning during summer, presence of nevi and presence of freckles. All subjects were of self-reported European ancestry.

### Whole-genome sequencing

Methods used for whole-genome sequencing, imputation and association analysis have been described previously (8,10–12). In this study, we used whole-genome sequence data derived from 2230 Icelanders sequenced to an average coverage of at least 10× using Illumina GAIIx and HiSeq2000 instruments at the deCODE Genetics facility. This detected ∼38.5 million SNP and small indel variants. All SNP and indel locations are given in NCBI Hg18 Build 36 coordinates.

### Illumina SNP chip genotyping

The Icelandic chip-typed samples were assayed with the Illumina HumanHap300, HumanHapCNV370, HumanHap610, 1M, or Omni-Quad bead chips at the deCODE Genetics facility. SNPs were excluded if they had (i) a yield of <95%, (ii) a MAF of <0.01 in the population, (iii) an excessive deviation from Hardy–Weinberg equilibrium (*P* < 10^−6^), (iv) an excessive inheritance error rate (>0.001) or (v) if there was a substantial difference in allele frequency between chip types (in which case, the SNP was removed from a single chip type if that resolved the difference, but if it did not then the SNP was removed from all chip types). All samples with a call rate of <97% were removed from the analysis.

### Imputation and association testing

We used imputation assisted by long-range haplotype phasing and genealogy-based *in silico* genotyping (8), to determine the genotypes of the 38.5 million variants for 4208 Icelanders with BCC (of whom 2726 were genotyped by Illumina chip and 1482 by genealogy-based *in silico* methods) and 109 408 control individuals (of whom 70 876 were genotyped by Illumina chip and 38 532 by genealogy-based *in silico* methods). For the HumanHap series of chips, 304 937 SNPs were used for long-range phasing, whereas for the Omni series of chips, 564 196 SNPs were used. An initial imputation step was carried out on each chip series separately to create a single harmonized, long-range phased genotype dataset consisting of 707 525 SNPs. Subsequently, this genotype dataset was used in the second step of imputing the full set of 38.5 million variants that were tested for association. Association testing was performed using logistic regression, matching controls to cases based on how informative the imputed genotypes were and correcting for familial relatedness using genomic control. For quality control purposes, association tests were carried out to screen out variants that showed frequency differences between the HumanHap and Omni chip platform series. Joint analysis of multiple case–control replication groups was carried out using a Mantel–Haenszel model in which the groups were allowed to have different population frequencies for alleles but were assumed to have common relative risks. Tests for heterogeneity were performed by comparing the null hypothesis of the effect being the same in all populations to the alternative hypothesis of each population having a different effect, using a likelihood ratio test. We also calculated the *I*^2^ statistic, which lies between 0 and 100% and describes the proportion of total variation in the study estimates that is due to heterogeneity (36). For conditional analyses, the allele count of each individual was given as a covariate in the logistic regression. Note that for conditional analyses, only chip-derived genotypes were used (i.e. we did not use genealogy-based *in silico* genotypes) and the results are unadjusted for genomic control.

### Centaurus single-track genotyping

All non-Icelandic samples and a subset of the Icelandic samples were genotyped using Centaurus single-track assays (15). Primer sequences for assays are available on request. For each SNP tested by single-track assay, all samples were genotyped at the deCODE Genetics facility. Clustering algorithms were applied, and manual editing was carried out in the same way for all samples. Two standard DNA samples and water blanks were included on every plate.

### Assessment for potential overlap with regulatory regions

For each region examined (chr20:2,158,646-2,258,645 for *TGM3* and chr8:101,064,000-101,170,000 for *RGS22*), we took the strongest signal (i.e. rs214782 and rs7006527 for *TGM3* and *RGS22*, respectively) and identified all SNPs and small indels in LD with it at *r*^2^ > 0.8 (excluding SNPs with low imputation information values). We then adjusted the *P*-value and OR of the strongest signal for the effect of each correlated SNP in turn. If the resulting *P*_adj_ was greater than an arbitrary threshold of 10^−4^, we considered that the correlated SNP in question could not be resolved statistically from the SNP showing the strongest signal. This yielded a set of 40 correlated variants for the *TGM3* locus and 24 variants for the *RGS22* locus. Note that in this procedure, the analysis is limited to SNPs and small indels that were identified by the whole-genome sequencing and which could be imputed with information values of ≥0.9, [see (8)]. Note also that we limited the search to the top signals at each locus, on the grounds that little resolution is achieved by this method unless the initial signal is reasonably strong. For each of the unresolvable variants identified, we searched ENCODE data (16,17) for physical overlaps between the variant location and suspected regulatory sites as follows: firstly, we used Ensembl to determine whether the variant location has been assigned an ENSR number. Secondly, we examined the UCSC hg19 data with particular emphasis on the “Integrated Regulation from ENCODE” supertrack set. Thirdly, we examined the data as presented in HaploReg v2 (37). And fourthly, we examined the relevant entry in RegulomeDB (38).

### Expression analysis

Samples of RNA from human peripheral blood were hybridized to Agilent Technologies Human 25K microarrays as described previously (18). We quantified expression changes between two samples as the mean logarithm (log_10_) expression ratio (MLR) compared with a reference pool RNA sample. In comparing expression levels between groups of individuals with different genotypes, we denoted the expression level for each genotype as 10^(average MLR)^, where the MLR is averaged over individuals with the particular genotype. We determined s.e.m. and significance by regressing the MLR values against the number of risk alleles carried. We took into account the effects of age, sex and differential cell type count in blood as explanatory variables in the regression. *P*-values were adjusted for familial relatedness of the individuals by simulation. For RT-PCR analysis, we converted total RNA (from a subset of the samples that were used for the microarrays) to cDNA using the High Capacity cDNA Archive Kit (Applied Biosystems), primed with random hexamers. We designed two assays covering exon–intron junctions in the *TGM3* gene and carried out quantitative PCR according to the manufacturer's instructions on an ABI Prism 7900HT Sequence Detection System. We assessed differences in mean relative abundance of TGM3 RNA between genotypic groups by regression, as described earlier.

## URLS

HaploReg v2, http://www.broadinstitute.org/mammals/haploreg/haploreg.php; RegulomeDB, http://regulomedb.org/index.

## SUPPLEMENTARY MATERIAL

Supplementary Material is available at *HMG* online.

## FUNDING

J.I.M. is supported by Red Tematica de Investigacion Cooperative en Cancer RD06/0020/1054. The Danish study “Diet, Cancer and Health” was supported by grants from the Danish Cancer Society and “Europe Against Cancer”: European Prospective Investigation into Cancer and Nutrition (EPIC). Funding to pay the Open Access publication charges for this article was provided by deCODE Genetics/AMGEN.

## AUTHORS’ CONTRIBUTIONS

The study was designed and the results interpreted by S.N.S., P.S., D.F.G., U.T., A.K., J.H.O. and K.S. Subject ascertainment and recruitment was carried out by S.N.S., B.S., K.R.B., K.T., R.R., V.F., C.C., M.G., D.P., O.S., P.R., E.G., K.K., K.H., B.A.N., A.T., K.O., U.V., R.K., E.N., J.I.M., T.R. and J.H.O. Sequencing, genotyping and expression analysis was done by S.N.S., A.J., G.T., G.M., J.G., H.J., H.T.H., U.T. and T.R. Statistical and bioinformatics analysis was done by S.N.S., P.S., D.F.G., G.T., S.A.G., G.M., J.G. and A.K. The manuscript was drafted by S.N.S., P.S., D.F.G., A.J., G.T., J.G, T.R. and K.S. All authors contributed to the final version of the paper. Principle collaborators for the case–control population samples were B.S. and J.H.O (Iceland), U.V. (Denmark), R.K. (Eastern Europe), E.N. (Valencia, Spain) and J.I.M (Zaragoza, Spain).

## Supplementary Material

Supplementary Data
