# Supplementary material for: Germline sequence variants in TGM3 and RGS22 confer risk of basal cell carcinoma
Source: Hum Mol Genet. 2014 Jan 8;23(11):3045–53. doi: 10.1093/hmg/ddt671 (PMC4014188; doi:10.1093/hmg/ddt671)
Supplement: Supplementary Data [file supp_23_11_3045__index.html]

Germline sequence variants in TGM3 and RGS22 confer risk of basal cell carcinoma — Germline sequence variants in TGM3 and RGS22 confer risk of basal cell carcinoma — Supplementary Data 

# Germline sequence variants in *TGM3* and *RGS22* confer risk of basal cell carcinoma

## Supplementary Data

Supplementary Data

**Files in this Data Supplement:**

- Supplementary Data - Docx file
