# Supplementary material for: Germline sequence variants in TGM3 and RGS22 confer risk of basal cell carcinoma
Source: Hum Mol Genet. 2014 Jan 8;23(11):3045–53. doi: 10.1093/hmg/ddt671 (PMC4014188; doi:10.1093/hmg/ddt671)
Supplement: Supplementary Data [file supp_ddt671_ddt671supp.docx]

Supplementary Material for: **Germline Sequence Variants in *TGM3* and *RGS22* Confer Risk of Basal Cell Carcinoma**

**Simon N. Stacey et al.**

| **Supplementary Table S1: Previously Published and Current GWAS Results for BCC Associated Variants^a^** | | | | | | | | | |  |  |
| --- | --- | --- | --- | --- | --- | --- | --- | --- | --- | --- | --- |
|  |  |  |  |  | **Published Result^b^** | | | | **Current Result** | | |
| **SNP** | **Allele** | **Chr** | **Position^c^** | **Locus^d^** | **OR** | **95%CI** | **P** | **Publication** | **OR** | **95%CI** | **P** |
| rs7538876 | A | 1 | 17,594,950 | PADI6/RCC2 | 1.27 | (1.15,1.41) | 1.9x10^-6^ | (6) | 1.25 | (1.18, 1.32) | 7.2x10^-14^ |
| rs801114 | G | 1 | 227,064,458 | RHOU | 1.32 | (1.20, 1.46) | 5.0x10^-8^ | (6) | 1.25 | (1.17, 1.32) | 2.4x10^-13^ |
| rs401681 | C | 5 | 1,375,087 | TERT | 1.27 | (1.19, 1.36) | 9.5x10^-12^ | (5) | 1.23 | (1.16, 1.30) | 2.2x10^-12^ |
| rs11170164 | T | 12 | 51,199,935 | KRT5 | 1.29 | (1.14, 1.46) | 4.4x10^-5^ | (7) | 1.25 | (1.14, 1.37) | 3.1x10^-6^ |
| rs2151280 | G | 9 | 22,024,719 | CDKN2A/B | 1.22 | (1.13, 1.31) | 7.6x10^-8^ | (7) | 1.20 | (1.14, 1.27) | 3.0x10^-10^ |
| rs157935 | T | 7 | 130,236,093 | KLF14 | 1.25 | (1.15, 1.35) | 3.6x10^-8^ | (7) | 1.23 | (1.15, 1.31) | 8.5x10^-11^ |
| rs78378222 | G | 17 | 7,512,477 | TP53 | 2.36 | (1.93, 2.89) | 5.2x10^-17^ | (8) | 2.24 | (1.90, 2.64) | 4.4x10^-22^ |
| rs214782 | G | 20 | 2,229,970 | TGM3 | 1.27 | (1.16, 1.39) | 1.8x10^-7^ | NA | 1.29 | (1.20, 1.38) | 3.1x10^-12^ |
| rs7006527 | C | 8 | 101,093,681 | RGS22 | 0.74 | (0.67, 0.82) | 9.6x10^-9^ | NA | 0.77 | (0.70, 0.83) | 9.0x10^-10^ |
| ^a^ association results obtained in the Icelandic population sample | | | | | |  |  |  |  |  |  |
| ^b^ for *TGM3* and *RGS22*, the result given is the one obtained at the time of reference (8), even though these results were not presented in that publication | | | | | | | | | | | |
| ^c^ NCBI HG18 Build 36 | |  |  |  |  |  |  |  |  |  |  |
| ^d^ locus named by nearby gene(s). This does not imply that the named gene necessarily mediates the pathogenic effect of the variant. | | | | | | | | | | |  |

| **Supplementary Table S2: Linkage Disequilibrium Values for Non-Icelandic Populations** | | | | |
| --- | --- | --- | --- | --- |
| **SNP1** | **SNP2** | **N^1^** | **D´** | **r^2^** |
| **Spain Zaragoza** |  |  |  |  |
| rs59586681 | rs214782 | 1999 | 0.27 | 0.01 |
| rs59586681 | rs214803 | 1988 | 0.27 | 0.01 |
| rs59586681 | rs214830 | 1987 | 0.03 | 0.00 |
| rs214782 | rs214803 | 2079 | 0.97 | 0.93 |
| rs214782 | rs214830 | 2070 | 0.09 | 0.00 |
| rs214803 | rs214830 | 2053 | 0.10 | 0.00 |
| rs7006527 | rs3133679 | 2020 | 1.00 | 0.14 |
| **Spain Valencia** |  |  |  |  |
| rs59586681 | rs214782 | 2017 | 0.35 | 0.02 |
| rs59586681 | rs214803 | 2227 | 0.37 | 0.02 |
| rs59586681 | rs214830 | 2199 | 0.01 | 0.00 |
| rs214782 | rs214803 | 2088 | 0.96 | 0.91 |
| rs214782 | rs214830 | 2044 | 0.04 | 0.00 |
| rs214803 | rs214830 | 2259 | 0.02 | 0.00 |
| rs7006527 | rs3133679 | 1938 | 0.98 | 0.14 |
| **Eastern Europe** |  |  |  |  |
| rs59586681 | rs214782 | 2017 | 0.35 | 0.02 |
| rs59586681 | rs214803 | 2227 | 0.37 | 0.02 |
| rs59586681 | rs214830 | 2199 | 0.01 | 0.00 |
| rs214782 | rs214803 | 2088 | 0.96 | 0.91 |
| rs214782 | rs214830 | 2044 | 0.04 | 0.00 |
| rs214803 | rs214830 | 2259 | 0.02 | 0.00 |
| rs7006527 | rs3133679 | 1938 | 0.98 | 0.14 |
| **Denmark** |  |  |  |  |
| rs59586681 | rs214782 | 2017 | 0.35 | 0.02 |
| rs59586681 | rs214803 | 2227 | 0.37 | 0.02 |
| rs59586681 | rs214830 | 2199 | 0.01 | 0.00 |
| rs214782 | rs214803 | 2088 | 0.96 | 0.91 |
| rs214782 | rs214830 | 2044 | 0.04 | 0.00 |
| rs214803 | rs214830 | 2259 | 0.02 | 0.00 |
| rs7006527 | rs3133679 | 1938 | 0.98 | 0.14 |
| ^1^Number of samples genotyped for both SNPs | | | |  |

| **Supplementary Table S3: Correlation between genotypes determined by imputation and Centaurus direct genotyping.** | | | |
| --- | --- | --- | --- |
| **SNP** | **Description in Text** | **Number Genotyped by Both Methods** | **Correlation Coefficient^a^** |
| rs214782 | Top | 2564 | 0.945 |
| rs214803 | T13K | 2956 | 0.952 |
| rs59586681 | Distal | 6770 | 0.968 |
| rs214830 | G654R | 5316 | 0.88 |
| rs7006527 | RGS22 | 11043 | 0.982 |
| ^a^ correlation coefficient determined by comparing genotypes imputed from chip-typed individuals with genotypes determined by Centaurus single-track assay. | | | |

| **Supplementary Table S4: Detailed Association Results for Variants in TGM3 and RGS22** | | | | | | |  |  |  |  |  |  |  |  |
| --- | --- | --- | --- | --- | --- | --- | --- | --- | --- | --- | --- | --- | --- | --- |
| **SNP** | **Allele** | **Chr** | **Position^a^** | **Locus** | **Description in Text** | **Sample Set** | **Number Cases** | **Number Controls** | **Frequency in Controls** | **OR** | **95%CI** | **P** | **P_heterogeneity_** | **I^2^** |
| rs214782 | G | 20 | 2,229,970 | TGM3 | Top | Iceland | 4208^b^ | 109,408^c^ | 0.171 | 1.29 | (1.20,1.38) | 3.1x10^-12^ |  |  |
|  |  |  |  |  |  | Spain Zaragoza | 315 | 1817 | 0.169 | 1.50 | (1.21,1.84) | 1.5x10^-4^ |  |  |
|  |  |  |  |  |  | Spain Valencia | 338 | 1757 | 0.184 | 1.32 | (1.08,1.61) | 0.0076 |  |  |
|  |  |  |  |  |  | Eastern Europe | 527 | 525 | 0.169 | 1.21 | (0.96,1.51) | 0.1 |  |  |
|  |  |  |  |  |  | Denmark | 300 | 310 | 0.189 | 1.12 | (0.82,1.52) | 0.47 |  |  |
|  |  |  |  |  |  | **Combined Non-Icelandic** | **1480** | **4409** |  | **1.31** | **(1.17,1.46)** | **3.5x10^-6^** | **0.38** | **2.9** |
|  |  |  |  |  |  | **All Combined** | **5688** | **113,817** |  | **1.29** | **(1.22,1.37)** | **5.5x10^-17^** | **0.54** | **0** |
| rs214803 | G | 20 | 2,238,333 | TGM3 | T13K | Iceland | 4208^b^ | 109,408^c^ | 0.172 | 1.27 | (1.18,1.37) | 3.9x10^-11^ |  |  |
|  |  |  |  |  |  | Spain Zaragoza | 315 | 1796 | 0.170 | 1.46 | (1.19,1.80) | 3.8x10^-4^ |  |  |
|  |  |  |  |  |  | Spain Valencia | 339 | 1995 | 0.181 | 1.33 | (1.09,1.62) | 0.0057 |  |  |
|  |  |  |  |  |  | Eastern Europe | 486 | 520 | 0.160 | 1.22 | (0.96,1.55) | 0.099 |  |  |
|  |  |  |  |  |  | Denmark | 294 | 299 | 0.184 | 1.19 | (0.87,1.61) | 0.27 |  |  |
|  |  |  |  |  |  | **Combined Non-Icelandic** | **1434** | **4610** |  | **1.32** | **(1.18,1.48)** | **2.2x10^-6^** | **0.61** | **0** |
|  |  |  |  |  |  | **All Combined** | **5642** | **114,018** |  | **1.28** | **(1.21,1.37)** | **5.0x10^-16^** | **0.72** | **0** |
| rs59586681 | T | 20 | 2,168,310 | TGM3 | Distal | Iceland | 4208^b^ | 109,408^c^ | 0.395 | 0.86 | (0.81,0.91) | 5.7x10^-7^ |  |  |
|  |  |  |  |  |  | Spain Zaragoza | 313 | 1715 | 0.365 | 0.84 | (0.70,1.01) | 0.063 |  |  |
|  |  |  |  |  |  | Spain Valencia | 331 | 1900 | 0.370 | 0.83 | (0.70,0.99) | 0.04 |  |  |
|  |  |  |  |  |  | Eastern Europe | 519 | 486 | 0.424 | 0.83 | (0.70,1.00) | 0.05 |  |  |
|  |  |  |  |  |  | Denmark | 291 | 285 | 0.382 | 0.98 | (0.72,1.33) | 0.9 |  |  |
|  |  |  |  |  |  | **Combined Non-Icelandic** | **1454** | **4386** |  | **0.85** | **(0.77,0.94)** | **0.0012** | **0.81** | **0** |
|  |  |  |  |  |  | **All Combined** | **5662** | **113,794** |  | **0.86** | **(0.82,0.90)** | **2.5x10^-9^** | **0.91** | **0** |
| rs214830 | G | 20 | 2,269,105 | TGM3 | G654R | Iceland | 4208^b^ | 109,408^c^ | 0.311 | 0.91 | (0.85,0.97) | 0.0024 |  |  |
|  |  |  |  |  |  | Spain Zaragoza | 309 | 1794 | 0.384 | 0.99 | (0.81,1.21) | 0.93 |  |  |
|  |  |  |  |  |  | Spain Valencia | 334 | 1932 | 0.381 | 1.00 | (inf,0.00) | 1 |  |  |
|  |  |  |  |  |  | Eastern Europe | 523 | 520 | 0.353 | 0.94 | (0.78,1.12) | 0.49 |  |  |
|  |  |  |  |  |  | Denmark | 300 | 297 | 0.352 | 0.86 | (0.68,1.09) | 0.22 |  |  |
|  |  |  |  |  |  | **Combined Non-Icelandic** | **1466** | **4543** |  | **0.94** | **(0.83,1.05)** | **0.27** | **0.86** | **0** |
|  |  |  |  |  |  | **All Combined** | **5674** | **113,951** |  | **0.91** | **(0.87,0.97)** | **0.0014** | **0.92** | **0** |
| rs7006527 | C | 8 | 101,093,681 | RGS22 | Top | Iceland | 4208^b^ | 109,408^c^ | 0.142 | 0.77 | (0.70,0.83) | 9.0x10^-10^ |  |  |
|  |  |  |  |  |  | Spain Zaragoza | 295 | 1747 | 0.139 | 0.74 | (0.56,0.97) | 0.031 |  |  |
|  |  |  |  |  |  | Spain Valencia | 313 | 1888 | 0.144 | 0.70 | (0.54,0.92) | 0.01 |  |  |
|  |  |  |  |  |  | Eastern Europe | 522 | 510 | 0.166 | 0.90 | (0.70,1.16) | 0.43 |  |  |
|  |  |  |  |  |  | Denmark | 297 | 297 | 0.172 | 0.71 | (0.51,0.99) | 0.042 |  |  |
|  |  |  |  |  |  | **Combined Non-Icelandic** | **1427** | **4442** |  | **0.77** | **(0.67,0.88)** | **2.3x10^-4^** | **0.5** | **0** |
|  |  |  |  |  |  | **All Combined** | **5635** | **113,850** |  | **0.77** | **(0.71,0.82)** | **8.7x10^-13^** | **0.67** | **0** |
| ^a^NCBI HG18 Build 36 | |  |  |  |  |  |  |  |  |  |  |  |  |  |
| ^b^Total number of cases used for association testing, including 2726 chip-genotyped and 1482 *in silico-*genotyped individuals | | | | | | | |  |  |  |  |  |  |  |
| ^c^Total number of controls used for association testing, including 70,876 chip-genotyped and 38,532 *in silico*-genotyped individuals | | | | | | | | | |  |  |  |  |  |

| **Supplementary Table S5: Effect of age on association of SNPs in *TGM3* and *RGS22* with basal cell carcinoma^a^** | | | | | | |
| --- | --- | --- | --- | --- | --- | --- |
|  |  |  | **Without age adjustment** | | **With age adjustment** | |
| **SNP** | **Allele** | **Locus** | **OR** | **P** | **OR** | **P** |
| rs214782 | G | TGM3 | 1.282 | 2.2x10^-14^ | 1.281 | 5.5x10^-15^ |
| rs59586681 | T | TGM3 | 0.867 | 1.3x10^-7^ | 0.877 | 4.4x10^-7^ |
| rs7006527 | C | RGS22 | 0.778 | 4.6x10^-11^ | 0.782 | 7.5x10^-11^ |
| ^a^ age data for cases and controls was available for Iceland, Spain Zaragoza, Eastern Europe and Denmark sample sets. | | | | | | |

| **Supplementary Table S6: Conditional analyses of SNPs associated with BCC at TGM3 and RGS22 loci** | | | | | | |  |  |  |  |
| --- | --- | --- | --- | --- | --- | --- | --- | --- | --- | --- |
| SNP A | | SNP B | | Sample Set | Association SNP A Unadjusted | | | Association SNP A Adjusted for SNP B | | |
| Name | Description | Name | Description |  | OR | 95%CI | P | OR_adj_ | 95%CI | P_adj_ |
| rs214803 | T13K | rs214782 | Top | Iceland | 1.28 | (1.20, 1.38) | 7.0x10^-13^ | 0.65 | (0.40, 1.04) | 0.073 |
|  |  |  |  | Combined Non-Icelandic | 1.31 | (1.17, 1.47) | 2.1x10^-6^ | 1.06 | (0.67, 1.67) | 0.81 |
|  |  |  |  | All Combined | 1.29 | (1.22, 1.37) | 8.0x10^-18^ | 0.84 | (0.60, 1.16) | 0.29 |
| rs214782 | Top | rs214803 | T13K | Iceland | 1.30 | (1.21, 1.39) | 5.4x10^-14^ | 2.00 | (1.24, 3.22) | 0.0043 |
|  |  |  |  | Combined Non-Icelandic | 1.30 | (1.16, 1.46) | 3.6x10^-6^ | 1.28 | (0.81, 2.02) | 0.29 |
|  |  |  |  | All Combined | 1.30 | (1.23, 1.38) | 1.0x10^-18^ | 1.58 | (1.14, 2.20) | 0.0062 |
| rs59586681 | Distal | rs214782 | Top | Iceland | 0.88 | (0.84, 0.94) | 1.9x10^-5^ | 0.90 | (0.85, 0.95) | 1.8x10^-4^ |
|  |  |  |  | Combined Non-Icelandic | 0.86 | (0.78, 0.94) | 0.0013 | 0.88 | (0.80, 0.96) | 0.0072 |
|  |  |  |  | All Combined | 0.88 | (0.84, 0.92) | 1.0x10^-7^ | 0.89 | (0.85, 0.93) | 4.5x10^-6^ |
| rs214830 | G654R | rs214782 | Top | Iceland | 0.92 | (0.87, 0.98) | 0.0083 | 0.92 | (0.86, 0.97) | 0.0048 |
|  |  |  |  | Combined Non-Icelandic | 0.96 | (0.87, 1.05) | 0.36 | 0.96 | (0.88, 1.05) | 0.38 |
|  |  |  |  | All Combined | 0.94 | (0.89, 0.98) | 0.0070 | 0.92 | (0.88, 0.98) | 0.0040 |
|  |  | rs214782 & rs59586681 | Top & Distal Jointly | Iceland | 0.92 | (0.87, 0.98) | 0.0083 | 0.93 | (0.88, 0.98) | 0.0079 |
| rs3133679 | RGS22 42%MAF | rs7006527 | RGS22 Top | Iceland | 0.94 | (0.89, 0.99) | 0.020 | 0.88 | (0.83, 0.93) | 7.0x10^-6^ |
|  |  |  |  | Combined Non-Icelandic | 0.91 | (0.83, 0.99) | 0.035 | 1.04 | (0.94, 1.15) | 0.43 |
|  |  |  |  | All Combined | 0.93 | (0.89, 0.97) | 0.0020 | 0.92 | (0.87, 0.96) | 5.0x10^-4^ |

| **Supplementary Table S7: ENCODE data for variants highly correlated with TGM3 rs214782 (*Top*)** | | | | | | | |  |  |
| --- | --- | --- | --- | --- | --- | --- | --- | --- | --- |
| **Marker Location** | **Name** | **r^2^ vs rs214782** | **D´ vs rs214782** | **Risk allele** | **Other allele** | **Freq. of risk allele** | **P_adj_^1^** | **Ensembl ENSR / ENST number** | **HaploReg Comments** |
| chr20:2229970 | rs214782 | 1.000 | 1.000 | G | A | 0.185 | NA | ENSR00000396606 | Weak Enh; DNaseHS in 21 cell types; 11 proteins bound ChIP: PU1, USF2, YY1, NFKB, FOSL2, CFOS, CJUN, JUNB, JUND, USF1, MAX |
| chr20:2230049 | rs214783 | 0.997 | 1.000 | A | C | 0.185 | 0.82 | ENSR00000396606 | Weak Enh; DNaseHS in 41 cell types; 10 proteins bound ChIP: PU1, USF2, YY1, NFKB, PAX5C20, CEBPB, USF1, CFOS, CJUN, JUNB |
| chr20:2229284 | rs214781 | 0.999 | 1.000 | A | C | 0.185 | 0.50 | null | Weak Enh; DNaseHS in 6 cell types; 1 protein bound ChIP CEBPB; CEBPA/B motif altered |
| chr20:2231667 | rs214787 | 0.967 | 0.997 | C | T | 0.188 | 0.13 | null |  |
| chr20:2234057 | rs214794 | 0.968 | 0.997 | G | A | 0.188 | 0.080 | null |  |
| chr20:2231684 | rs214788 | 0.949 | 0.997 | G | A | 0.192 | 0.070 | null |  |
| chr20:2228615 | rs214778 | 0.957 | 0.985 | A | G | 0.187 | 0.064 | null |  |
| chr20:2233035 | rs214792 | 0.967 | 0.997 | G | A | 0.189 | 0.059 | null |  |
| chr20:2232264 | rs214789 | 0.947 | 0.997 | A | G | 0.192 | 0.057 | null |  |
| chr20:2232906 | rs214790 | 0.964 | 0.997 | G | A | 0.189 | 0.048 | null |  |
| chr20:2233898 |  | 0.965 | 0.997 | AGTCACTG | A | 0.190 | 0.037 | null |  |
| chr20:2234704 | rs214799 | 0.967 | 0.997 | T | G | 0.189 | 0.034 | ENSR00001446644 | Weak Enh; DNAseHS in 5 cell types; 2 proteins bound ChIP: FOXA1, FOXA2 |
| chr20:2233705 | rs214793 | 0.964 | 0.997 | C | T | 0.190 | 0.031 | null |  |
| chr20:2231457 | rs214785 | 0.970 | 0.997 | C | T | 0.189 | 0.027 | null |  |
| chr20:2234191 | rs214795 | 0.969 | 0.997 | C | G | 0.189 | 0.024 | null |  |
| chr20:2234343 | rs214796 | 0.968 | 0.997 | C | A | 0.189 | 0.022 | null |  |
| chr20:2226992 | rs214768 | 0.979 | 1.000 | A | G | 0.183 | 0.021 | null |  |
| chr20:2230665 | rs214784 | 0.971 | 1.000 | C | T | 0.189 | 0.020 | null |  |
| chr20:2223528 | rs214758 | 0.981 | 1.000 | C | T | 0.182 | 0.013 | null |  |
| chr20:2232974 | rs214791 | 0.970 | 0.997 | C | T | 0.189 | 0.010 | null |  |
| chr20:2227081 | rs214769 | 0.981 | 1.000 | G | A | 0.183 | 0.0096 | null |  |
| chr20:2225892 | rs214762 | 0.983 | 1.000 | T | C | 0.183 | 0.0094 | null | Weak Enh; DNAseHS in 2 cell types |
| chr20:2223976 | rs214760 | 0.983 | 1.000 | C | T | 0.183 | 0.0075 | null |  |
| chr20:2235003 | rs66612109, rs151040643 | 0.970 | 0.997 | AAGA | A | 0.189 | 0.0075 | null |  |
| chr20:2226028 | rs214763 | 0.983 | 1.000 | G | A | 0.183 | 0.0061 | null |  |
| chr20:2222810 | rs214757 | 0.984 | 1.000 | G | A | 0.183 | 0.0049 | null |  |
| chr20:2222381 | rs214756 | 0.977 | 0.998 | G | A | 0.182 | 0.0047 | null |  |
| chr20:2238333 | rs214803 | 0.967 | 0.995 | C | A | 0.188 | 0.0043 | ENST00000381458 T13K |  |
| chr20:2228391 | rs214776 | 0.982 | 1.000 | G | A | 0.183 | 0.0037 | null |  |
| chr20:2228300 | rs214774 | 0.984 | 1.000 | C | T | 0.183 | 0.0027 | null |  |
| chr20:2227894 | rs214772 | 0.975 | 0.997 | C | T | 0.183 | 0.0027 | null |  |
| chr20:2227909 | rs214773 | 0.980 | 1.000 | C | T | 0.183 | 0.0026 | null |  |
| chr20:2228355 | rs214775 | 0.984 | 1.000 | T | C | 0.183 | 0.0019 | null |  |
| chr20:2226674:2 |  | 0.976 | 0.997 | - | AAC | 0.184 | 0.0018 | null |  |
| chr20:2218015 | rs214748 | 0.977 | 0.998 | C | T | 0.183 | 9.1x10^-4^ | ENSR00000396601 | Weak promoter; Strong Enh |
| chr20:2221987 | rs214755 | 0.981 | 1.000 | A | T | 0.183 | 8.2x10^-4^ | null |  |
| chr20:2227435 | rs214770 | 0.983 | 1.000 | A | G | 0.183 | 6.2x10^-4^ | ENSR00001446642 |  |
| chr20:2236590 | rs214801 | 0.962 | 0.992 | G | A | 0.188 | 2.5x10^-4^ | null |  |
| chr20:2240273 | rs214807 | 0.881 | 0.963 | G | A | 0.179 | 1.1x10^-4^ | null |  |
| chr20:2210537 | rs6082600 | 0.809 | 0.995 | C | T | 0.157 | 1.1x10^-4^ | null |  |
| ^1^P value for residual effect of rs214782 when adjusted for indicated SNP | | | | | |  |  |  |  |

| **Supplementary Table S8: ENCODE data for variants highly correlated with RGS22 rs7006527 (*Top*)** | | | | | | | | |  |
| --- | --- | --- | --- | --- | --- | --- | --- | --- | --- |
| **Marker Location** | **Name** | **r^2^ vs rs7006527** | **D´ vs rs70026527** | **Risk allele** | **Other allele** | **Freq. of risk allele** | **P_adj_^1^** | **Ensembl ENSR / ENST number** | **HaploReg Comments** |
| chr8:101093681 | rs7006527 | 1.000 | 1.000 | C | A | 0.143 | NA | null |  |
| chr8:101071860 | rs28622940 | 0.983 | 1.000 | A | G | 0.142 | 0.99 | null |  |
| chr8:101070462 | rs28671717 | 0.968 | 0.996 | A | T | 0.141 | 0.84 | null | MAFK bound ChIP in 1 cell type |
| chr8:101105528 | rs7004782 | 0.993 | 1.000 | A | G | 0.143 | 0.77 | null |  |
| chr8:101068813 | rs28758904 | 0.985 | 1.000 | A | G | 0.142 | 0.76 | null |  |
| chr8:101116624 | rs28884287 | 0.996 | 1.000 | T | C | 0.143 | 0.72 | null |  |
| chr8:101076800 | rs138516187 | 0.984 | 1.000 | T | C | 0.142 | 0.69 | null |  |
| chr8:101094807 | rs16897870 | 0.997 | 1.000 | T | C | 0.143 | 0.68 | null |  |
| chr8:101132064 | rs7818948 | 0.996 | 1.000 | A | G | 0.143 | 0.64 | null |  |
| chr8:101131314 | rs10099237 | 0.995 | 1.000 | C | G | 0.143 | 0.62 | null |  |
| chr8:101127489 | rs10104624 | 0.995 | 1.000 | C | G | 0.143 | 0.59 | null |  |
| chr8:101091916 | rs12334713 | 0.994 | 1.000 | T | C | 0.143 | 0.54 | null |  |
| chr8:101076513 | rs113174200 | 0.985 | 1.000 | C | A | 0.142 | 0.50 | null |  |
| chr8:101113970 | rs144035626 | 0.994 | 1.000 | C | T | 0.143 | 0.48 | null |  |
| chr8:101076845 | rs28792460 | 0.965 | 0.989 | G | A | 0.145 | 0.46 | null |  |
| chr8:101101375 | rs10046693 | 0.982 | 0.996 | A | G | 0.142 | 0.46 | null |  |
| chr8:101105011 | rs28410444 | 0.995 | 1.000 | A | T | 0.143 | 0.46 | null |  |
| chr8:101074196 | rs141115006 | 0.964 | 0.989 | T | C | 0.144 | 0.37 | null |  |
| chr8:101097415 | rs6989943 | 0.994 | 1.000 | A | G | 0.143 | 0.36 | null |  |
| chr8:101124722 | rs34338006 | 0.993 | 1.000 | TC | T | 0.143 | 0.26 | null |  |
| chr8:101080524 | rs2199333 | 0.984 | 1.000 | T | C | 0.142 | 0.088 | null |  |
| chr8:101126763 | rs3116089 | 0.953 | 1.000 | A | C | 0.149 | 0.049 | null |  |
| chr8:101160745 | rs10111694 | 0.914 | 0.975 | A | T | 0.139 | 0.0013 | null |  |
| chr8:101165265 | rs113394050 | 0.893 | 0.971 | C | T | 0.137 | 7.38x10^-4^ | ENSR00001397532 |  |
| ^1^P value for residual effect of rs7006527 when adjusted for indicated SNP | | | | | | |  |  |  |
